# An agent based model representation to assess resilience and efficiency of food supply chains

**DOI:** 10.1371/journal.pone.0242323

**Published:** 2020-11-19

**Authors:** George Van Voorn, Geerten Hengeveld, Jan Verhagen

**Affiliations:** Wageningen University & Research, Droevendaalsesteeg, Wageningen, The Netherlands; Uniiversity of Padova, ITALY

## Abstract

Trying to meet the Sustainable Development Goals is challenging. Food supply chains may have to become more efficient to meet the increasing food requirement of 10 Billion people by 2050. At the same time, food and nutrition security are at risk from increasingly likely shocks like extreme climate events, market shocks, pandemics, changing consumer preferences, and price volatility. Here we consider some possibilities and limitations regarding the improvement of resilience (the capacity to deal with shocks) and efficiency (here interpreted as the share of produced food delivered to consumers) of food supply chains. We employ an Agent Based Model of a generic food chain network consisting of stylized individuals representing producers, traders, and consumers. We do this: 1/ to describe the dynamically changing disaggregated flows of crop items between these agents, and 2/ to be able to explicitly consider agent behaviour. The agents have implicit personal objectives for trading. We quantify resilience and efficiency by linking these to the fraction of fulfilment of the overall explicit objective to have all consumers meet their food requirement. We consider different types of network structures in combination with different agent interaction types under different types of stylized shocks. We find that generally the network structures with higher efficiency are also more sensitive to shocks, while less efficient network types display more resilience. At first glance these results seem to confirm the existence of a system-level trade-off between resilience and efficiency similar to what is reported in business management and ecology literature. However, the results are modified by the trading interactions and the type of shock. In our simulations resilience and efficiency are affected by ‘soft’ boundaries caused by the preference and trust of agents (i.e., social aspects) in trading. The ability of agents to switch between trading partners represents an important aspect of resilience, namely a capacity to reorganize. These insights may be relevant when considering the reorganization of real-life food chains to increase their resilience to meet future food and nutrition security goals.

## Introduction

The projected increase in population to 10 Billion people in 2050, of which 68% is projected to live in urban areas, poses serious challenges to food and nutrition security—defined by FAO as “when all people, at all times, have physical, social and economic access to sufficient, safe and nutritious food which meets their dietary needs and food preferences for an active and healthy life” [1]. There is an increasing demand for the production and delivering of food, feed, fiber, fuel, etc.; a challenge that has been captured in Sustainable Development Goal (SDG) 2: zero hunger [2]. Currently we do not meet the food requirement [3, 4], and hence it is necessary to further improve the (efficiency of the) output of food supply chains, e.g., by avoiding waste, improving nutrient recycling, and decreasing yield gaps. At the same time, food supply chains should be considered within a larger scope as food systems, involving not only the different (possibly international) actors from farmer to consumer, but including all processes and infrastructure involved in getting a population fed, such as providing inputs, growing, harvesting, processing, transporting, selling, consuming, and producing waste, within environmental, social, economic, and political contexts, and dependent on human labour. Moreover, the spatial boundaries of these systems are not always clear, and they furthermore are under stress from an increasing scarcity of inputs and available land, soil degradation, and climate change [3, 5, 6]. This may increase their vulnerability to shocks, such as weather extremes, economic shocks, pandemics, and political uproar, even up to a level at which a collapse may occur. This is not unthinkable, as food systems have collapsed in the past as a result of over-exploitation. One example is the North-Atlantic cod fishery [7], a food system that had largely shaped the communities of Canada’s eastern coast for centuries until the fish stocks crashed. In addition, food systems have evolved into large, complex, and international networks, linking consumers to producers across countries and continents [8]. An analysis of a directed network that represents crop trade connections between countries during the period 1992-2009 reveals that the global food system is relatively homogeneous and highly connected [9]. There are increasingly tight interconnections between the terrestrial livestock, crop, fishery, and aquaculture sectors [10]. There is also a strong dependence of food systems on ecosystem services that are not directly included, such as pollination, biological control, carbon sequestration, and soil improvement [11]. An analysis of historical food trade data from 1986 through 2013 reveals there is an annual increase in the connectivity, and it is unclear how the increased interconnectedness and the network structure of current food systems affect their vulnerability to shocks [12]. The connectedness of different food systems may result in new vulnerabilities [13]: events such as the banking crisis in 2008 and its aftermath suggest that interconnected systems may experience cascading effects, i.e., the collapse of one system (‘the weakest link’) may result in a ‘domino effect’ with far-reaching consequences [14–16]. Social and ecological drivers, influenced by the dynamics of the food system, can spill over multiple food sectors and create synchronous challenges or trade-offs among terrestrial and aquatic systems, as illustrated by [17]. They further stress that achieving the SDGs by 2030 will require addressing drivers of food production shocks and derived threats.

Summarizing, there is a need to increase the output of food systems to meet SDG2, but at the same time there may be an increased probability of food system collapse under an increasing demand for food system services needed to meet global demand. In other words, there are limitations to food system services that may be in sight under the current regime. The question then is: Are we already at the edge of what is feasible, and if not, what can be done to progress the increase of food system services while keeping risks of failure at an acceptable level, or preferably at the same time even decrease them?

In order to address this question, we need to describe food supply chains and food systems quantitatively. These systems do not respond in a (near) linear way to their drivers. In particular when considering food systems, we have to consider including the relevant processes associated with food security, including the natural, economic, technological, and cultural environments [18, 19]. They are hence complex systems, in which people interact with each other and with their natural environment for delivering food services. I.e., they consist of trade and social networks of different human agents who try to satisfy their personal objectives, like farmers, fishers, consumers, companies, NGOs, governments, etc., and whose decisions affect each other and their environment and vice versa through various interactions [20, 21]. In this paper, we adopt a stylized view of food systems as food supply chains, i.e., complex systems in which flows are enabled from source agents (like producers) to sink agents (like consumers); these flows may not only concern goods, but also services and information [22]. We include key actors involved in food production, trading, and consumption, where different types of shocks, possibly from outside the direct food supply chain, affect the dynamics between these actors.

Importantly, the complex and nonlinear nature of food systems also implies they contain mechanisms that generate capacity for absorbing pressure and shocks. This concept is known in the literature as ‘resilience’, and has received considerable attention in the ecological and socioecological literature [6, 23], but which has to be defined in the context of food systems here. The classic view of resilience is the time it takes for the system to restore after the shock has disappeared [24], assuming the system has a steady state to return to. However, resilience not only has ‘biophysical’ components (e.g., soil chemical buffers), but also ‘social’ and ‘organizational’ components that are equally important [25]. Humans can display the ability to adapt to new situations in a short time interval. They interact in dynamic social and economic networks through which they can learn from past experience and respond to the decisions and actions of others. Also, human agents are not always rational and are bounded by social norms, emotions, and limited perceptions, which may affect their adaptability, e.g., their willingness to adopt more sustainable practices [26]. The ability of the individual to change may in turn also change the vulnerability of the larger food systems to shocks. This adaptive capacity is a key aspect of resilience [27, 28]. In fact, it has been argued that resilience may even be better conceptualized as adaptability than stability, as under some circumstances stability may actually point to a failure to adapt to change [29]. One may adopt a view of food systems as living organisms with defense mechanisms, which need to be challenged in order to develop (which can be roughly translated as “what does not kill us, makes us stronger”). It is therefore more prudent to describe the resilience of food systems as “the capacity of [the food system] to absorb disturbance and reorganize while undergoing change so as to still retain essentially the same function, structure, identity, and feedbacks” [6, 27].

An important part of the adaptive capacity of actor-based food supply chains may reside in the ability of agents to link to other agents in the supply chain. I.e., food supply may run through different alternative ‘pathways’ from producers to consumers. These alternatives may be limited in, e.g., current food supply chains centered around consumers in the Western world, which seem to be pushed towards optimized efficiency, and are organized as ‘hourglass-type’ networks consisting of many consumers and producers, but few intermediate agents [30]. Another example of a seemingly optimized supply chain with limited pathways is presented by the Dutch pig sector, where the number of farms has halved in the period from 2000 through 2010 while the number of pigs per farm doubled. With constantly increasing competition, lowering prices per kilo pork and stable political conditions, farmers lowered their costs and increased their efficiency at the cost of the environment, animal welfare and operational flexibility [31]. On the one hand, it shows there is adaptability among farmers to respond to drivers, becoming more efficient as individuals, while at the other hand the question can be raised, how much further this development can go before the situation is untenable in terms of food system resilience. Trade-offs between efficiency and resilience are for instance recognized in the field of business management [32, 33], and equally apply to food systems, which consists of managed businesses, and even on multiple levels (like individual farms and country level). An increased efficiency (the share of produced food delivered to consumers) may possibly come with a reduction of the resilience (i.e., the capacity to deal with future shocks and restore system services) because mechanisms for buffering and adaptation, such as the existence of alternative pathways, may have been eroded.

In this paper we address this apparent trade-off between efficiency and resilience as part of the larger question how we can meet the increased demand for food system services while keeping vulnerabilities to shocks in check. The main focus is on the underlying networks of trade involving different (classes of) agents, through which services (like crop production) are delivered to consumers. Although we know there is a plethora of processes involved in food systems [34, 35] of which several may involve relevant resilience mechanisms, it seems sensible to start with a generic, relatively simple network model involving the agents who are the primary decision makers and who may change their behaviour in response to shocks (for instance, by relinking to other agents), as it represents a considerable part of the capacity of the system to become more efficient or to absorb shocks and reorganize. For example, a network would be less vulnerable to shocks when the loss of a few nodes would not lead to a significant loss of transfer of information across the network [30]. However, such a network may be less efficient than a network with fewer nodes, since splitting the trade flow over multiple nodes has a lower probability of bringing the information from the source to the end point. An additional complication may reside in the ability of agents in the network to change, which in turn may affect the resilience and efficiency of the network. Our research question is, whether there is indeed a dominant trade-off between efficiency and resilience, or there is possibly a way to optimize food systems (represented as trade networks) so they are both sufficiently efficient and resilient to meet supply demands?

In the following we first present an Agent Based Model to represent the main agents in a generic food trade network. We select this modelling framework to allow for the inclusion of explicit mathematical descriptions of disaggregated trade interactions between agents as well as their social and behavioural mechanisms. No specific case study has been selected, because we are primarily interested here in the question of what common mechanisms may affect resilience and efficiency (the model is intended to be expanded in future work to include case-specific particularities). We then present an experimental design, and introduce the metric to quantify resilience and efficiency, which is based on information theory. In this paper no data are yet used for model calibration and validation. We then present the main results, and conclude the paper with a discussion of the key findings and suggestions for follow-up research.

## Agent based model

Experimentation on food systems is difficult and even unethical for several obvious reasons. The development and use of a model allows for the explicit quantitative testing of hypothesized processes and mechanisms. We develop an Agent Based Model (ABM) in order to explicitly include rules of social behaviour and decision-making of agents, the interaction network of agents, and the diversity within the agent population(s) and changes therein, while still being able to include system dynamics [21, 36, 37]. We consider a generic food system, i.e., the model includes main features shared by most if not all food systems, without focusing on the specific characteristics of any particular food system. Note, that in the current model system dynamics (like crop growth and weather processes) have been excluded for simplicity. We realize the generic nature and the exclusion of system dynamics limits the applicability of this model study for specific applications, but again, we are mainly focusing on the trade network structure. Different model settings are explored in a numerical experimental design to generate simulations, which we use to determine what networks are more resilient than others. Moreover, in the context of resilience it has to be clarified “resilience of what to what?” [38]. Hence, several different shocks are included in the numerical design to which the simulated agents can respond. The model coded in NetLogo [39] is available via OpenABM (https://www.comses.net/codebases/05f6bc80-f79e-4850-9066-ffd914395aa4/releases/1.0.0/).

For the description of the model we loosely use the ODD format for the documentation of ABMs [40].

### Purpose of the model

This ABM is intended to represent a generic, dynamic, hierarchical food system network of producers, traders, and consumers. Each model simulation is in fact a trading game that includes a series of production cycles, while in each production cycle several trading cycles are embedded. The ABM is used for studying the trade-off between efficiency and resilience of food supply systems by looking at different network and agent interaction types in relation to different shocks. The food supply chain functioning is evaluated based on the average level of ‘satisfaction’ of the consumers.

### Entities and state variables

The ABM contains a social network in which three types of agents interact. Each agent type represents a different role in the food system:

- Producers, who at the start of each production cycle produce either of two ‘crops’ (the model is flexible in the number of crops it can handle, but we set it to two by default), and who sell these items to traders based on active selection (see interaction modes further below);
- Traders, who passively accept crops from producers (currency is not explicitly included in the current model code) and passively ‘display’ crops for selection by consumers;
- Consumers, who actively select traders from whom to buy. Each iteration they try to fulfil their individual dietary needs regarding the existing types of crop.

The main variables per agent type are given in Table 1.

**Table 1:** Main variables per agent type.

Agent type	Variable	Description	Type	Temporal dynamics
**Producer**	Crop_type	The crop type currently produced	Int	Each iteration
	Production	The volume of the crop currently produced	Double	Changes during specific shock period
	Production memory	The volume of crop produced at the start (to be able to get back after shock)	Double	Fixed
	My_trader	Trader to whom current production is sold	Agent	Each trade iteration
	My_historictraders	List of traders to whom crops have been sold in the past	Agent set	Accumulates each trade iteration (no loss of memory)
**Trader**	Producerset	Current producers from whom is bought	Agent set	Each trade iteration
	Consumerset	Current consumers to whom is sold	Agent set	Each trade iteration
	Product quantitylist	Amount of crop in store >per crop type	Double [NCroptypes]	Each trade iteration
**Consumer**	Consumption QuantityList	Amount of crop consumed per croptype per period this tick	Double [nPeriods,NCroptypes]	nPeriods per Tick
	Demandlist	Amount of crop required per period per croptype	Double [nPeriods,NCroptypes]	Changes with specific shock period
	Demandlistmemory	To be able to recover after a shock	Double [nPeriods,NCroptypes]	Fixed
	MyTrader	Trader currently bought from	Agent	nPeriods per Tick
	MyHistoricTraders	List of traders from whom crops have been	Agent set bought in the past	Accumulates

Computer program variables used by the different agent types.

### Basic principles, process overview and scheduling

**Fig 1:**
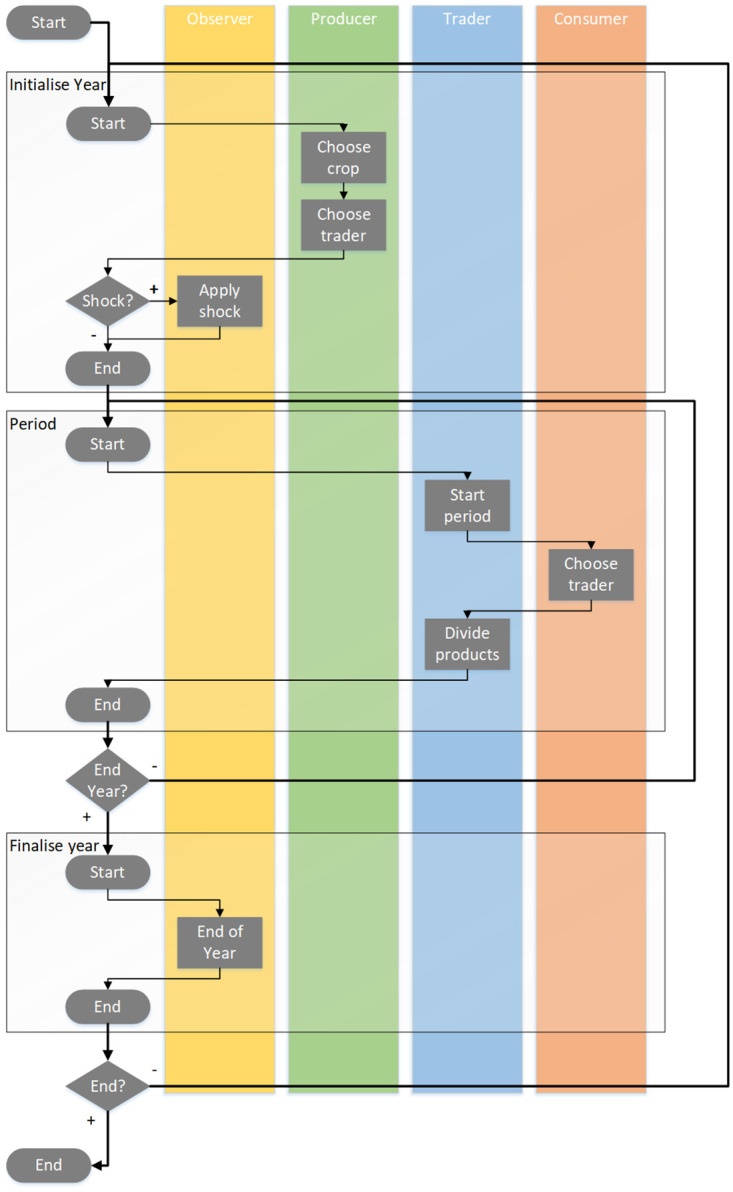
Flowchart of the model. The ABM consist of a level of producers (in green), traders (in blue), and consumers (in coral); ‘observer’ (light orange) deals with user-defined decisions and actions. Agents select partners to trade with. Traders also have the ability to stock to sell later. ‘Year’ refers to production cycle. ‘Period’ refers to consumption cycle.

The ABM represents a generalized food system as an abstracted economic game in which crops are produced, traded, and consumed, taking place in a non-spatial, social network of agents. Agents attempt to procure crops in indirect competition. A graphical conceptual overview of the generic model is given in Fig 1.

Several key model assumptions set this ABM apart from most economic models. First, there is no assumption of equilibrium between supply and demand. Food supply is consistently lagging behind food requirement [4], i.e., the minimal nutritional and caloric needs of all consumers will not be met. This is why in this model we set a default value for crop production that is lower than the default value of the requirement. Second, while price formation is an important driver of trade, price is not explicitly included in this model. Instead, in the *weighted* interaction mode (see Subsection on ‘Interactions’ below), we assume that traders divide their products equally between multiple consumers ‘buying’ from them, while consumers search for the trader(s) with the largest stock, implicitly assuming that the trader with the highest stock will automatically offer the lowest price. Vice versa, producers look for traders with lowest stocks with the assumption they will offer higher prices. In other words, agents behave according to economic utility, but in the ABM the relative availability of crops is taken as proxy for the relative price traders ask. No explicit monetary stocks and flows are modelled. Untraded crops remain in stock for possible trade in consecutive iterations, although the parameter setting can be changed to include a loss term from storage. These assumptions present an important reason why the ABM is a dynamic model instead of an equilibrium model.

The order of actions by the different agents is as follows. Each production cycle (‘year’) all producers choose a crop, and produce it. Each producer chooses a trader to sell their produced crop to (based on indirect economic utility, and possibly modified, see the below Subsection on ‘Interactions’). Each consumption cycle (interpreted as ‘month’ when the parameter for the number of consumption cycles is set to ‘12’) trading takes place. Each consumer chooses a trader to buy from (again based on indirect economic utility). Trading interactions are direct. We assume a strictly hierarchical, three-level layered network structure, in which the agents are nodes, and the trading with crops forms the unidirectional flow links (again, the money flow in the reverse direction is in this model formulation only implicit), in which each link represents a connection from agent *i* to *j*, and has time-dependent attributes like capacity and flow rate [22]. For our analysis we assume that agents share a link if they (commonly) trade during some time interval—and, hence, no link exists if within that time interval no crops are exchanged from agent *i* to *j*. Producers only trade with traders, who in turn only trade with consumers, and no direct trade between producer and consumer can take place. Traders are therefore a necessary intermediate in the flow of crops from producers to consumers. Traders split the crops they have in stock evenly between the different consumers buying from them, up to the requirement of these consumers. Left-over crops are stocked for the next consumption cycle. The trading network is organized according to one of several pre-defined types (discussed further below). Trading takes place according to one of three possible interaction modes: random, according to basic economic utility optimization, or a limited economic utility optimization, constrained by preference for known traders (also discussed further below).

Further important model assumptions are:

- Links between individual agents can change in time according to one of the three (below-described) interaction types, i.e., links between agents can (dis)appear during a simulation;
- The number of agents of each type remains fixed during a simulation, and agents do not switch agent type, i.e., they keep their place in the agent network. Agents cannot disappear from a simulation, even if they would go ‘bankrupt’ or do not fulfil their dietary needs (this assumption is justified here as we are interested in the impact of shocks, and not in the exact development of the food system in time), and no new agents are introduced during a simulation.

Simulations take 300 iterations, of which the first 100 present a ‘start-up’ phase, and the next 100 the ‘pre-shock’ phase. After 200 iterations a shock period takes place in which one of three possible shocks occurs that lasts 100 iterations. The severity of the shock is determined as a severity parameter (set to 0.5 by default) times the number of randomly selected agents of the level at which the shock is applied to. The three potential types of shock are:

- Shock at the producer level, which could e.g. be interpreted as ‘failed harvest’. A fixed percentage of producers—determined by the severity parameter—will not produce crops. This reduces the total volume of crops available to traders to buy;
- Shock at the trade level, which could e.g. be interpreted as ‘stock loss’. A fixed percentage of traders—determined by the severity parameter—will lose their complete stock. This reduces the total volume of crops available to traders to sell;
- Shock at the consumption level, which could, e.g., be interpreted as ‘preference change’. A fixed percentage of consumers—determined by the severity parameter—will shift their preference from one crop type to another. This changes their objective to fulfil their individual requirement, and changes the requirement of consumers of what to buy from traders.

### Scales

The ABM does not have an explicit spatial scale. Although agents are shown at specific locations in the simulation environment, this does not mean there are explicit spatial processes. Instead, there is only a social network of producers, traders, and consumers, where agents have a ‘position’ in the network without an explicit spatial location. The implicit assumption following from this is there are no costs for transport. Although the agent typology implies agents to be individuals, agents can also be interpreted as companies, super-individuals, or even countries in their roles of producing / exporting and consuming / importing crops. The temporal scale is set to 1 production cycle and 12 consumption cycles (‘months’) per tick, mimicking a yearly cycle with a single production of crops each year, in which consumers can change supplier each month. The difference in temporal scales represents the higher flexibility of consumers for choosing where and when to buy, then for producers when to sell. In the analysis the effects of stochasticity are dampened by the use of the average over a large numbers of iterations.

### Interactions

There are three interaction modes one can select from when initializing the model (i.e., once the interaction mode has been set, it keeps this mode during simulation):

- The *random* mode. This entails the random selection of a partner. There are no explicit decisions but only random pairing of producer with trader and trader with consumer. This also means trading is **not** according to economic principles. This mode presents a ‘baseline’;
- The *weighted* mode. Selection occurs according to ‘best interest’, i.e., agents evaluate which of the possible trading partners offers the best price for buying or selling, respectively. As mentioned above, price optimization is implicit. Producers select traders negatively weighted by the traders’ stock of the crop the producer is producing, mimicking the highest selling price. Consumers select traders positively weighted by the traders’ stock of the crop for which the cumulative fulfilment of the requirement is lowest, mimicking the lowest buying price. Selection is based on perfect information, which is a common—yet admittedly doubtful—assumption in economic theory. Excess crop volume is stocked by traders;
- The *preference* mode. Selection is based on a combination of perceived best price and a preference based on trading history. This is hence similar as for the weighted mode, but now those traders with whom has been traded in previous production and consumption cycles get additional weight in the selection process. This interaction mode mimics price sensitive behaviour constrained by preference for trusted partners. This is a reasonable assumption, as trust plays an important role in trading.

Also, one can select from seven possible networks (see Table 2), divided over four different types:

- Block type, i.e., there are equal numbers of producers, traders, and consumers;
- Inverse pyramid type, i.e., there are few producers, more traders than producers, and more consumers than traders;
- Hourglass type, i.e., there are more producers and consumers than traders;
- Diamond type, i.e., there are more traders than producers and consumers.

**Table 2:** Network types included in the numerical experiment.

Network type	Network code	No. of producers	No. of traders	No. of consumers	No. of possible links
**Block (small)**	bs	5	5	5	50
**Block (large)**	bl	20	20	20	800
**Inverse pyramid (small)**	is	5	25	50	1375
**Inverse pyramid (large)**	il	10	50	100	5500
**Hourglass**	h	25	5	50	375
**Diamond (small)**	ds	5	50	25	1500
**Diamond (large)**	dl	10	100	50	6000

Columns including abbreviations (network code), number of producers, traders, and consumers, and the number of possible links.

All three interaction types and all seven networks have been included in the numerical experimental design (see next Subsection).

### Objectives

We distinguish between two types of objectives. All agent types (and hence all individual agents) have *implicit personal* objectives when trading. They (implicitly) aim to buy or sell at the best price (or a combination of best price and preference, explained further in the next Subsection). This applies to producers, traders, and consumers. Note, that the price dynamics are not explicitly included in this model, but we make the assumption that ‘price’ is determined implicitly by the size of the stock the trader has available. Producers sell off their product to traders who offer the best price, which is based on the trader having low stocks, which implies he offers a good price. Consumers buy at the lowest price. This is based on the trader having high stocks, which implies he is willing to accept a lower price. Traders do not ‘need’ to sell, and may ‘choose’ to store crop items for later transactions. These (implicit) ‘decisions’ are the result of a failure to link to a consumer in a certain iteration. The *explicit system-level* objective we consider in our analysis is the fulfilment of nutritional requirements, and in line with how we define efficiency (the share of produced food delivered to consumers). Consumers have the objective to fulfil their individual nutritional and food preference requirement, which is translated as a requirement that is set as a parameter for each crop type each cycle (while, as said before, they implicitly attempt to do so at the best price, or a combination of best price and preference). If they do not manage to fulfil these demands, their satisfaction drops (note, that in the model we now assume there is no starvation, bankruptcy, or any other adverse affects from failure to fulfil the requirement). During simulations the food system performance is evaluated through the fulfilment of consumer dietary needs: consumer satisfaction is measured as a discrepancy between what a consumer needs of each crop type each production cycle and what he manages to obtain via trading. Note, that because there is an inherent imbalance between supply and demand (i.e., shortage) in the model, on average consumers will not achieve 100% satisfaction (but individual consumers may do so). The satisfaction is calculated as the minimum over the crop types of the ratio of consumption to requirement (explained further in the next Subsection).

### Emergence

The model is used to study the quantification of resilience to shocks in food system networks. The networks are formed through the exchange of crops. Although the number of agents in a simulation is ‘fixed’ and producers cannot directly trade with consumers, the network of exactly who trades with whom emerges through these exchanges of crops, depending on the interaction rules. The resilience of the food system emerges from the capability of agents to change with whom they trade, and the capacity to take stock. Resilience is assessed as the difference in flows of crop volume between the shock and pre-shock period (explained further in the next Subsection). These flows are approximated by averaging the total crop volume traded between two agents over one of these two periods. Average flows differ between model configurations, determined by network type, interaction type, and shock type. Network types determine the ‘hard’ boundaries for the capacity to redirect flows. Interaction types determine the ‘soft’ boundaries, for instance, some potential links may exist but are never created due to preference interactions. The emergent property that is modelled is the (relative change in) resilience of the different food system network types and interaction modes in response to different shocks.

### Adaptation

The main source of adaptivity in this model is the capacity of agents to trade with other agents. Rules for trading depend on the interaction type. With random interaction selection the adaptation comes from the random making and breaking of trading links. The resilience is determined by the ‘hard’ boundaries, as in principle all potential links can be made (note also, that because the pairing is random trading is usually not optimized, and there is no penalty for poor fitness in this model, as agents cannot disappear from the simulation). If a shock results in the cutting of links, the random pairing ‘fixes’ this. With non-random interactions there are ‘soft’ boundaries to the generation of trading links. Links that would be feasible in theory (with random pairing) are now practically infeasible, which implies a reduced adaptation capacity. This is particularly the case for the preference interaction mode.

### Learning

There is no specific learning, other than that agents ‘remember’ with whom they have traded and assign weights to that in case of preference interactions.

### Prediction

Agents predict optimal trades in case of weighted or preference interactions.

### Sensing

Agents have perfect information on crop availability and ‘sense’ what traders have available. Agents also ‘sense’ their own internal states (the available crop, their weights to other agents). Interactions include ‘full’ information disclosure about these states to (potential) trading partners.

### Stochasticity

The model incorporates two sources of randomness:

- Each production cycle the producers are assigned a random crop type to produce;
- The selection of traders by producers and consumers is a random or a weighted random process.

To avoid dominance of model behaviour by stochasticity all other parameters, variables and processes are approximated to be fixed for the entire population or period.

### Initialization

The model includes 16 parameters that need to be set at initialization. The use of some of these is conditional on the value of others. These parameters are shown in Table 3. At initialization all agents are placed in the field. Producers are assigned a fixed production. Consumers are assigned a crop type each consumption cycle, depending on what is available.

**Table 3:** Parameters of the model.

Parameter	Description	Default value	Units
**N_Producers**	The number of producers	[1, →〉	(Agents)
**N_Traders**	The number of traders	[1, →〉	(Agents)
**N_Consumers**	The number of consumers	[1, →〉	(Agents)
**TotalProduction**	The total volume of crops produced by all producers combined per production cycle	100	Volume
**TotalDemand**	The total requirement by consumers for all crops combined per production cycle	120	Volume
**N_CropTypes**	The number of crop types in the simulation	2	–
**StockPersistence**	Percentage of stocked crops that persist into the next production cycle	0.95	Tick^−1^
**Periods**	Number of consumption cycles per production cycle	12	Tick^−1^
**SpinupPeriod**	Number of production cycles taken for spin up of the model (no output produced)	100	Ticks
**StationaryPeriod**	Number of production cycles taken as the standard situation	100	Ticks
**ShockDuration**	Number of production cycles the shock is applied	100	Ticks
**ChoiceModel**	The used interaction mode	Random / Weighted / Preference	–
**BasePreference**	Value used as basic weight for all traders for weighted and preference interaction mode	0.01	–
**ExpPreference**	Value used as weight for known traders in preference interaction mode	100	–
**ShockType**	Type of shock	Producer / Trader / Consumer	
**ShockSeverity**	Severity of the shock / share of population of targeted agents affected	0.5	–

The parameters of the model with description, default values and units. Parameters included in the sensitivity analysis are indicated in grey. Time units are set in Ticks, the default time setting in NetLogo, with the arbitrary interpretation of a year. The parameters included in the Sensitivity Analysis are indicated in grey.

### Observation

Output is discussed in detail in the next Subsection. The simulation runs are saved and all data from it are available, i.e., no data limitations or added noise have been considered to mimic real-life data properties.

### Input data

The model represents an iconic, generalized food system that is not calibrated to a particular real-life food system. No specific input data is used.

## Model analysis and quantifying resilience

The model is analyzed by using a numerical study design involving the three qualitative input factors (interaction mode, network, and shock type) and an OFAT (One Factor at A Time) Sensitivity Analysis involving continuous parameters. Each involved simulation consists of 300 iterations: 100 iterations for initialization, followed by 100 iterations pre-shock period, and then 100 iterations with the system under shock.

The main model output (of which all symbols are also given in Table 4) consists of the impact (effect) of a factor or parameter on the ability of consumers to fulfil their dietary needs. This (in)ability of consumers to fulfil their objective regarding the acquisition of crops links to the definition of food security and SDG2. The output is defined as

$$\begin{array}{c} E=\frac{{\bar{H}}_{p_{2}}-{\bar{H}}_{p_{1}}}{{\bar{H}}_{p_{1}}}\;. \end{array}$$ (1)

where *p*_1_ stands for the pre-shock period (StationaryPeriod), and *p*_2_ for the shock period (ShockDuration). The nested variable

$$\begin{array}{c} {\bar{H}}_{p_{.}}=\frac{\sum_{y}\min(\frac{c_{x,y}}{d_{x,y}})}{N\_Consumers} \end{array}$$ (2)

Here *x* is the index involving N_CropTypes crops between which consumers can choose; *y* iterates over all consumers, and *c*_*x*,*y*_ is the consumption and *d*_*x*,*y*_ the requirement for crop *x* by consumer *y*, respectively. The default value N_CropTypes = 2 is used in the simulations.

**Table 4:** Output symbols.

Symbol	Equation(s)	Description
*A*	(4)	Efficiency
*B*	(4)	Resilience
*C*	(4),(5)	Total capacity
*c*_*x*,*y*_	(2)	Consumption of crop *x* by consumer *y*
*d*_*x*,*y*_	(2)	Requirement of crop *x* by consumer *y*
*E*	(1)	Relative impact of shock (%)
*F*_*i*,*j*_	(4),(5)	Flow of information from *i* to *j*
*F*_*i*,._	(4)	Flow from *i* to all sinks
*F*_.,*j*_	(4)	Flow from all sources to *j*
*F*.,.	(4),(5)	∑_*i*,*j*_ *F*_*i*,*j*_
*H*	(1),(2)	Fulfilment of consumer objective
*i*	(4),(5)	Index for source compartment
*j*	(4),(5)	Index for sink compartment
*k*	(3)	Index for model input for sensitivity
*p*_1_	(1)	Index for pre-shock period
*p*_2_	(1)	Index for shock period
*q*_*k*_	(3)	Model input for sensitivity (e.g., Periods)
*r*	(3)	Index for combinations of inputs for model set-up
*S*_*k*,*r*_	(3)	Sensitivity of *Y*_*r*_ to *q*_*k*_
*x*	(2)	Index for crop types (N_CropTypes)
*y*	(2)	Index for N_Consumers
*Y*_*r*_	(3)	Model output for sensitivity (e.g., *E*)

Symbols used for the description of output, ordered alphabetically.

We run all combinations of the three qualitative input factors, which gives 3 × 7 × 3 = 63 possible combinations (the interaction mode has random, weighted, or preference-based, the seven networks are given in Table 2, and shock type can be at the production, trader, or consumer level). Because agent interactions are randomly initialized, we run 100 replicates per combination to account for stochasticity, giving 6300 simulation runs in total for the study design.

The OFAT analysis [41] involves continuous parameters which are varied step-wise across their range in sufficiently small steps, with one simulation per parameter setting to limit the computational costs of the numerical experiment. The results are visualized as scatterplots, plotting some output—for instance, impact *E* calculated by Eq (1)—as function of a parameter *q*. Sensitivities are then approximated as

$$\begin{array}{c} S_{k,r}=\frac{\Delta Y_{r}}{\Delta q_{k}}\;. \end{array}$$ (3)

Here index *k* indicates one of the six parameters involved in the OFAT analysis, namely BasePreference, ExpPreference, Periods, ShockSeverity, StockPersistence, and TotalDemand (see Table 3). The index *r* refers to the combination of the factors interaction mode (three options) and network (four options), and the level (producer, trader, or consumer levels), resulting in 36 different combinations (and hence as many scatterplots) for each parameter. To make the results of the OFAT analysis comprehensible, we classify the effects of parameters according to the sign of *S*_*k*,*r*_ and the qualitative effect on output *Y*_*r*_. That is, we report an on average negative, stable, or positive trend in the scatterplot as a minus (−), zero (0) or plus (+), respectively. Moreover, if all 36 scatterplots follow roughly the same pattern, we report only once for this one output. In cases the scatterplots require more attention, we discuss the results in more detail.

Efficiency and resilience are calculated by using the metric proposed by [42]. This metric has several advantages over the commonly used metrics for resilience, in particular the ‘return time’. Primarily, it captures both efficiency and resilience simultaneously, where resilience is interpreted as adaptive capacity, i.e., the capacity to reorganize. Additionally, the metric is calculated from the current system setting and does not require a shock to make the resilience evaluation, as is the case when the ‘return time’ is used. The metric is given as

$$\begin{array}{c} C=A+B\;;\;A=\sum_{i,j}F_{i,j}\log(\frac{F_{i,j}F_{.,.}}{F_{i,.}F_{.,j}})\;;\;B=-\sum_{i,j}F_{i,j}\log(\frac{{\left(F_{i,j}\right)}^{2}}{F_{i,}F_{.,j}})\;. \end{array}$$ (4)

Here *F*_*i*,*j*_ is the flow of information from compartment *i* to *j*, *F*.,. = ∑_*i*,*j*_ *F*_*i*,*j*_, *F*_*i*,_ is the flow from *i* to all sinks, and *F*._,*j*_ is the flow from all sources to *j* (where information can for instance be matter, energy, economic units, or, in this case, traded crops). Using algebraic rules for logarithms we can rewrite *C* as

$$\begin{array}{c} C=-\sum_{i,j}F_{i,j}\log(\frac{F_{i,j}}{F_{.,.}})\;. \end{array}$$ (5)

Quantity *C* is the system capacity. We interpret *A* as efficiency (the share of produced food delivered to consumers). In case a system configuration has the highest value of *A* compared to alternative configurations, it is maximally efficient in the transfer of crops from the start of the food chain to the consumers. *B* is interpreted as resilience, i.e., the capacity of the system structure to be reorganized so that a flow from some starting point to some end point is maintained after a shock. If *B* = 0, there is only a single link from start to end. If this link is cut, the transfer is reduced to zero, i.e., there is zero capacity to reorganize. On the other hand, a high value of *B* means there are alternative flows. The loss of a single link will then have little effect, at the cost of lower efficiency. If we assume that *C* remains unchanged (there is a fixed crop production over time), an increase in *B* implies a decrease in *A*, and vice versa. Note, that in case of a production shock, crop production and hence *C* drops.

In its original application, the metric is used with models of dynamical system equations in steady state, with fixed flows between compartments representing species and abiotic attributes like detritus [42]. In our ABM links between individual agents constantly appear and disappear during a simulation. We therefore calculate each of the flows *F*_*i*,*j*_ as an average of the flow of crops from agent *i* to agent *j* across a time window. When there is no link between two agents (which is a common situation in simulations), the flow between them is set to zero (note that log(0) mathematically is not defined). Because the model has developed to a sort of quasi steady state after a ‘spin-up’ period, we average across 100 production cycles. In the (S1 and S2 Figs) we show that this assumption holds better as the network is larger, because the effects of ‘noise’ then become smaller.

## Results

### Impact of different factors

**Fig 2:**
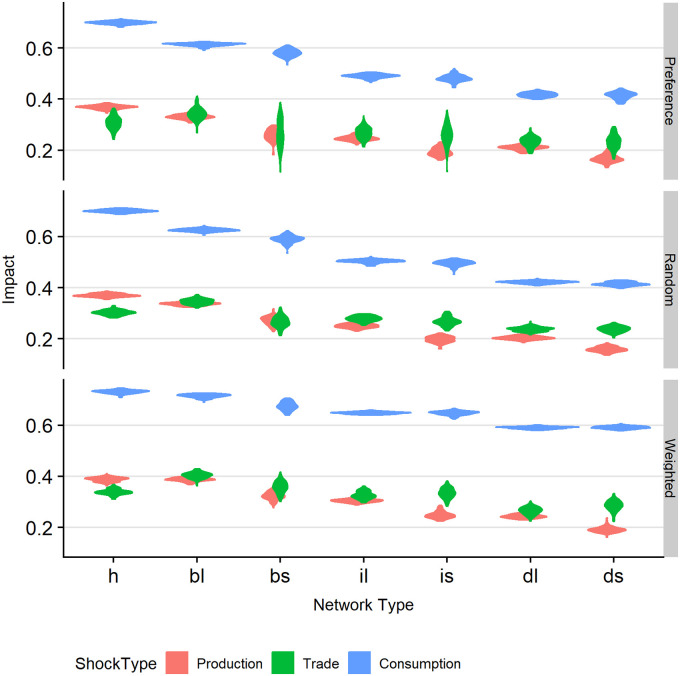
Triple-factor violin plot of impact affected by network type, shock type, and interaction type. Impact is defined by Eqs (1) and (2). Upper panel: preference interactions. Middle panel: random interactions. Lower panel: weighted interactions. Shock type is identified by colour: shock at production level (coral), shock at trade level (green), shock at consumption level (blue). Columns indicating network are ordered by decreasing impact. Networks: hourglass (h), large block (bl), small block (bs), large inverse pyramid (il), small inverse pyramid (is), large diamond (dl), and small diamond (ds).

Fig 2 summarizes the main results of the numerical experiment. Impact *E* calculated by Eqs (1) and (2) is depicted as function of shock type (by colour), network (by column), and interaction mode (by row). It should be noted that impact is relative. There is never 100% overall satisfaction, as supply has been set to lag requirement, although individual consumers may achieve a high level of satisfaction. Consumers in networks that display a large impact of shock usually also have a larger ability to fulfil their dietary needs before the shock (as we will see below). The impact of shocks is that the overall satisfaction decreases further. To cover the stochastic variability we display the results in a three-factor violin plot. Such a plot is similar to a box plot, but with a density representing the distribution of the output variable. I.e., a diamond-like shape indicates more variability in the output than a horizontally ‘smeared out’, pancake-like shape. Hence, a violin plot gives more information than only the summary statistics, as in the case of a box plot.

The first observation is that the impact of shocks on the consumption level is much higher than that of shocks on the trading or production level. This shock type is associated with changes in consumer preference for specific crops and the response time needed by producers and traders. In other words, the impact is a time-lag effect, resulting from the fact that producers need to switch their production, and traders need to build stocks with the new crop. In the case of shocks at the trading level the smaller networks display more variation in impact than their larger counterparts. This seems understandable, as smaller networks simply consist of fewer agents and hence display more noise, which will particularly show when shocks work on the intermediate network level, with more connections (namely to both producers and consumers).

The second observation is that with weighted interactions there is higher impact than with preference or random interactions. This may be explained from the observation that having agents with weighted interactions results in a more efficient network (i.e., the transfer of crops from producers to consumers is faster) than for the other interaction types, but simultaneously such a network has less resilience. Weighted interactions means that agents are searching for the best deal according to the well-known principle of economic utility optimization (‘*Homo economicus*’), and as a result the consumers on average are more efficient in fulfilling their dietary needs. As a drawback, shocks also have a higher impact with weighted interactions than with random or preference interactions. In the case of random interactions, links are randomly created and broken, and commonly enough links will be formed that are not optimal or even ‘bad’. As a result, the efficiency is lower, but so is the impact: severed links are also easily replaced by alternatives through the randomization. Preference interactions are also less optimal in terms of economic utility, as many potential links may not be formed because of the preference. I.e., agents prefer trusted partners over cheaper ones, which reduces the efficiency. Again, the impact of the shock is hence also lower.

**Fig 3:**
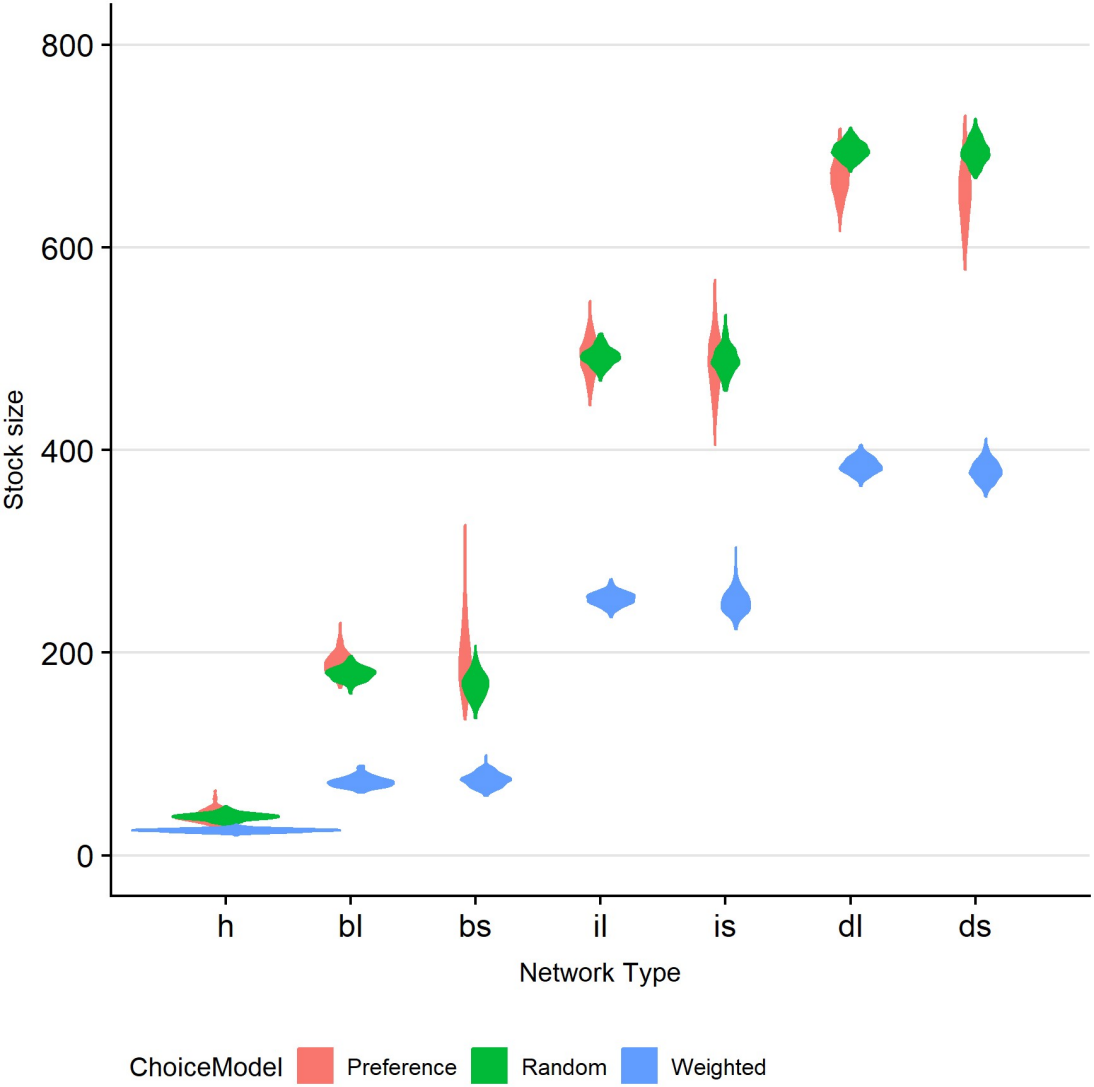
Accumulated stock sizes as function of network and interaction type. Interaction type is identified by colour: preference (coral), random (green), weighted (blue). Columns indicating network type are ordered by increasing stock size. Network types: hourglass (h), large block (bl), small block (bs), large inverse pyramid (il), small inverse pyramid (is), large diamond (dl), and small diamond (ds).

The supposed loss in efficiency may be supported by Fig 3. This Figure, again as violin plot, displays the stock size as function of network (by column) and interaction mode (dubbed ‘Choice Model’; by colour). The stocks under weighted interactions are much lower than those under random or preference interactions, suggesting a higher efficiency or better, a lower turn-over time with this interaction mode.

The third observation is that impact varies per network type. In Fig 2 the impact per network is displayed in descending order. The impact in the hourglass-type network (i.e., fewer traders than producers and consumers) is largest, except for the shock at the trading level. This network type is the most efficient, as can also be concluded from Fig 3. Likely many crops are quickly traded from producer to trader to consumer, as it is a matter of choice, i.e., there are relatively few traders to choose from. On the other hand, the diamond-type networks display the smallest impact, but also the highest stocks. These networks have more traders than producers and consumers, which seems to make them also relatively inefficient. The benefit of this inefficiency is the lower vulnerability to shocks.

Combined, the smallest impact occurs in diamond-type networks with random or weighted interactions and with shocks on the production level. In this case stocks are high while there is high inefficiency. Shocks have a smaller effect as there are many fallback options, namely alternative agents to trade with and/or plenty of stocks. The highest impact occurs in hourglass-type networks with weighted interactions and with shocks on the consumer level. The high efficiency and low stocks imply few fallback options and hence a high vulnerability to shocks, in particular with the delayed response time in the case of consumer shocks.

### Network information properties

**Fig 4:**
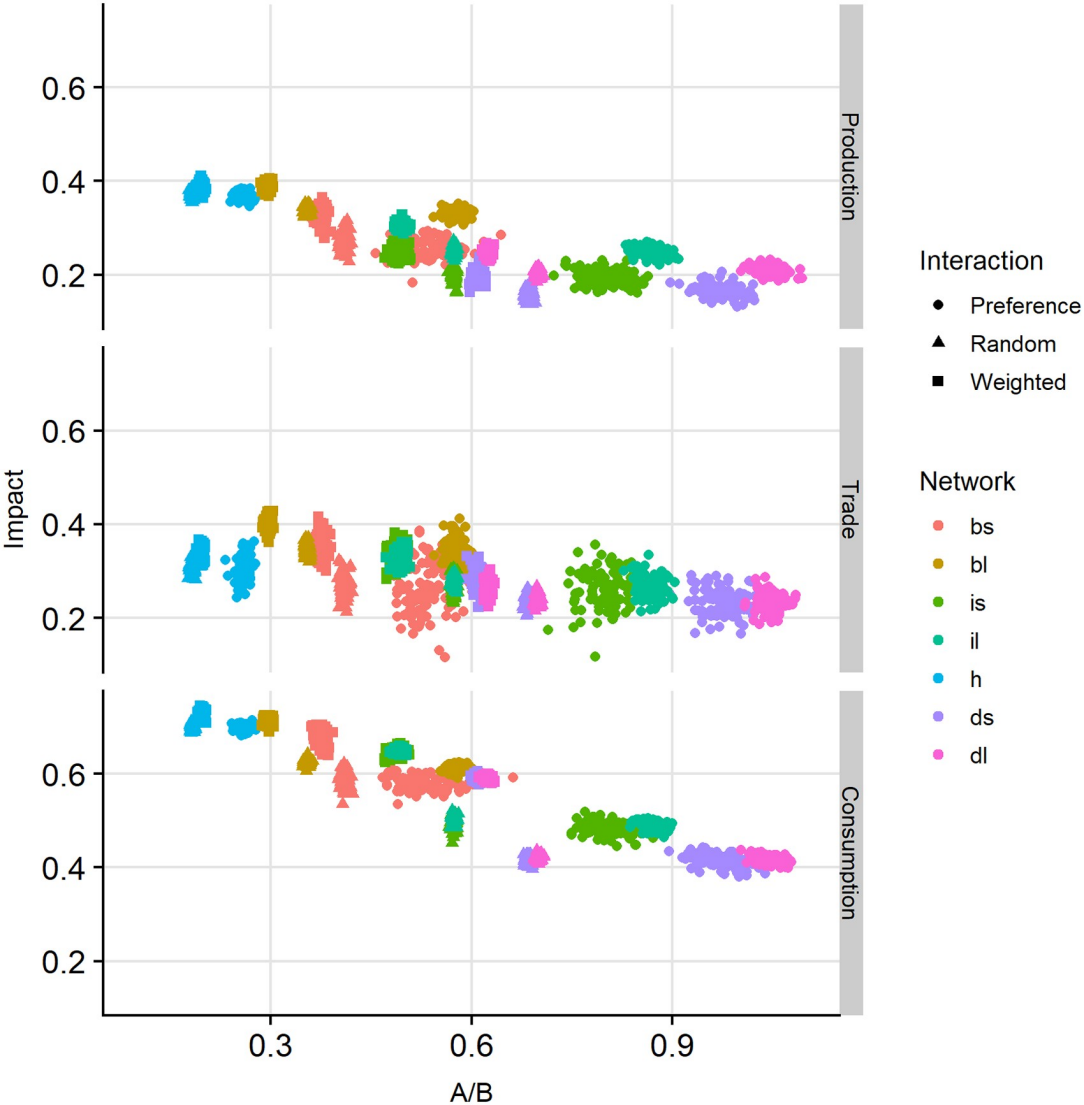
Impact of shock as function of ratio *A* to *B*, pre-shock period. Impact is defined by Eqs (1) and (2). We can interpret *A* as efficiency, and *B* as resilience. By taking the ratio of *A* to *B*, this plot indirectly shows the trade-off between *A* and *B* in the impact of extreme events. Upper panel: shock at production level. Middle panel: shock at trade level. Lower panel: shock at consumer level. Interaction type is identified by shape: preference (solid circle), random (solid triangle), and weighted (solid square). Network type is identified by colour: small block (coral), large block (golden brown), small inverse pyramid (green), large inverse pyramid (cyan), hourglass (blue), small diamond (purple), and large diamond (pink).

Fig 4 displays a scatterplot of the impact as function of the pre-shock efficiency *A* divided by resilience *B*, as calculated using Eq (4). The three panels are separated based on shock type, while the point colour indicates network and point shape the interaction mode. The upper panel gives the simulation output for simulations with production shock, the middle for trade shock, and the lower for consumption shock.

Points are mostly clearly clustered. Hourglass-type networks (blue) have the lowest ratio *A*: *B*, with a minimum value of around 0.17. Next are the block-type networks (coral and golden brown), with a minimal *A*: *B* = 0.3. The inverted pyramid-type networks (green and cyan) have minimal *A*: *B* = 0.45. Finally, the diamond-type networks (purple and pink) have minimal *A*: *B* = 0.6, while the maximum is above 0.9. This suggests that the *A*: *B* ratio could be an indicator for resilience, where a small value suggests the network is more efficient, and a larger value that it is more resilient against shocks. This measure is however not straightforward. The efficiency in hourglass networks is the result of having a limited number of routes for goods to be moved, and stocks remain low. The resilience in less efficient networks is partly the result of having many potential trading routes, i.e., there is a capacity to continue food trade through alternative links. At the same time, not all of these routes are used simultaneously, and stocks are built up. These stocks form another part of the resilience, i.e., a reserve that can supply if food production is disrupted.

The highest *A*: *B* ratio for each network type occurs for preference interactions (solid circles). This coincides with high stocks for this type of interactions. With random interactions the stocks are equally high, yet the *A*: *B* ratio is much closer to the ratio with weighted interactions for each network. This may be the result of strongly tied trading pairs in preference interactions, which means that during simulations also over longer time intervals many potential links are never formed. This results in many zero-links in the calculations of Eq (4) and hence a low value of *B*, in turn increasing the *A*: *B* ratio. With random interactions links are formed randomly, hence many of the potential links are in fact formed during longer time intervals. This generates many small flows, which increases the value of *B*, but with poorer efficiency than that achieved with weighted interactions.

### OFAT sensitivity analysis

The results of the OFAT analysis are displayed in Table 5.

**Table 5:** One factor at a time analysis results.

	BasePreference	ExpPreference	Periods	ShockSeverity	StockPersistence	TotalDemand
**Relative *A***	0	0 or +	0	0	0 or +	0 or −
**Relative *B***	0	0 or −	0	0	−	+
*C*	0	0 or −	0	0	+	0 or −
*H*_*p*1_	0	0	0	0	+	−
*H*_*p*2_	0	0	0	−	+	−
*H*_*p*1_ − *H*_*p*2_	0	0	0	−	−	+
**Impact *E***	0	0	0	−	0	0
**Stock**	0	0	0	0	+	−

Summarized results of the OFAT analysis. Trends in the scatterplots are indicated by sign (−, 0, or +). Grey refers to (near) zero sensitivities. In the main text more details are given.

The OFAT results can be summarized as follows:

1. **BasePreference** gives no clear, relevant effects. This is to be expected for the runs involving random or weighted interactions. We do not expect significant interaction effects. Although this parameter is needed in the coding for preference interactions, we conclude that the exact value of it is not important.
2. **ExpPreference** gives some quantitative effects only in the case of preference interactions. Nevertheless, these effects seem negligible. We conclude that also the exact value of this parameter is not important.
3. **Periods** is the parameter that indicates the number of trade cycles between consumers and traders compared to the crop production cycle. There are some quantitative effects, but only for Periods <5. It is not surprising that several trade cycles are required for consumers to acquire their desired goods. We conclude that for our default value of Periods = 12 the results are robust.
4. **ShockSeverity** has no qualitative effects on the quantities calculated with Eq (4) or the stock. It does however affect the output related to the ability of consumers to fulfil their dietary needs; in particular, the larger the value of this parameter, the larger the effect. This hardly seems surprising. We conclude that although there is a clear quantitative effect of this parameter, the qualitative results probably do not change.
5. **StockPersistence** is a parameter that affects almost all output. Values near 0 can be interpreted as representing perishable goods, while values near 1 represent non-perishable goods. The effects on stock are to be expected, as the larger the value, the larger the stock can grow. The effects on different quantities for calculating *E* are understandable, as the capability of consumers to fulfil dietary needs decreases with lower stock, i.e., lower persistence of stock. A value of 0 means no stocks can be built up, and $H_{p_{1}}$ and $H_{p_{2}}$ approach zero in that case. Interestingly enough, the effect on impact *E* is mostly negligible, probably as a result of counteracting effects: the impact is lower relatively speaking when the system before the shock also had lower stocks. Of particular interest is the observation that the hourglass network shows the least impact of all network types, unless weighted interactions are involved, in which case all networks reasonably approximate the response curve of the hourglass network. This suggests that the weighted interaction mode ‘evolves’ a hourglass-type network within the ‘hard’ boundaries of the larger network, resulting in a relatively successful setting for consumers to fulfil their dietary needs. Another interesting finding is that for large values of StockPersistence, *E* in diamond-type and inverted pyramid-type networks under production shocks considerably decreases. This suggests that these networks might be relatively resilient against production shocks as far as non-perishable goods are concerned.
6. **TotalDemand** affects not all output quantities, but in case they are affected, it is understandable, and in particular for stock, as a higher requirement implies stronger competition for the available goods and hence fewer left-over goods for traders to stockpile. The parameter shows additional effects on *C* and $H_{p_{1}}$ (and to a lesser extent also $H_{p_{2}}$) in the case of weighted interactions. In that case the other network types are closer to or even approach the response curve of the hourglass network. This may be explained in the same way as we explain the similar observations for StockPersistence: the weighted interaction mode leads to a *de facto* hourglass-type network within the larger network. In terms of *E* there are however no obvious effects from changes in this parameter.

## Discussion

In this paper we developed and analyzed an ABM to describe a generic food supply system, with the aim of quantifying food system efficiency (the share of produced food delivered to consumers) and resilience (its capacity to deal with shocks) to address the research question on the potential trade-off between efficiency and resilience, or the possibility to optimize food systems (represented as trade networks) so that they are both efficient and resilient. This research question is part of a bigger research topic on whether we can increase food system services while minimizing risks of failure of food systems in view of the SDGs. The model development reported here presents a first step, in which first principles of food chains have been used to develop the model and in which we compare the model output qualitatively to real-world systems. This limits the impact of our findings, but there are nevertheless some results that may be relevant to consider.

We have defined our main model output as ‘impact’ of shocks in the context of the ability of consumers to fulfil their food requirements. We use an information theory metric to quantify efficiency and resilience as terms of total system capacity [42], which makes the trade-off explicit. After a shock, the ‘impact’ shows the relative effect on consumer satisfaction, i.e., how much worse the consumers overall are off. Shocks on the production and trader level seemingly have a comparable impact (i.e., a comparable loss of available volume of crop items), but the underlying shock effect is different. In the case of production shock, several producers produce no crops; traders still have their stock, but may not be resupplied (or at least, at a lower rate). In the case of trader shock, several traders lose their stocks, presenting a sudden ‘leakage’, but production continues and so new crop items become available. The difference can be amplified by, e.g., changing the parameter ‘StockPersistence’ (as given in the OFAT analysis in Table 5). Shocks on the consumer level are more impactful than those on the other levels, as there is a lag-time effect from adaptation at the production side of the supply chain. More efficient networks also seem to show a larger impact, i.e., the effect of the shock is larger and apparently the resilience to cope with the shock is then less than that of less efficient networks. These networks trade away their crops faster, i.e., more efficiently, which increases the overall satisfaction of consumers, but also builds lower stocks. These stocks are however part of the resilience, i.e., the capacity to deal with the shock and still ‘feed’ the consumers. Another part of resilience is the capacity to form new trade links to available traders. In more efficient networks there are fewer intermediate traders, reducing the number of options for relinking after disruption. A plot of the ‘impact’ as function of the ratio A-to-B (efficiency to resilience) from the Ulanowicz metric shows that in particular in the case of shock at the consumer level the networks with the highest impact (i.e., highest relative effect of the shock) also have the lowest ratio A-to-B. This suggests this ratio could be used as a proxy for expected impact, but we argue there are more aspects that are not properly captured (e.g., behavioural aspects of agents in the trade network). We discuss our findings and model limitations in a broader context below.

First, we remark that the highest impact (loss of ability of consumers to fulfil their requirement) in our model occurs in the hourglass-like network with weighted interactions. These networks are the most efficient in terms of throughput of crops from producers to consumers of the networks that have been considered, and lead to the highest fulfilment of consumers. On the other hand, they also display the largest decrease in this fulfilment when there is a disruption. In less efficient networks the fulfilment by consumers is lower, but as a positive side-effect larger stocks are built by traders which serve as buffer when a disruption occurs. This appears to be a trade-off between efficiency and resilience similar to what has been reported in for instance the business economy literature; [33] point to such a trade-off and remark that the ‘leaning out’ of company operations also leads to limitations in dealing with possible disruptions, while [32] refer to the ‘*Ripple effect*’, as a goal for companies to develop recovery strategies aimed at compensating disruptions and thus avoid a ‘rippling’ through the supply chain. One avenue for future research is to consider looking at shorter supply chains, rather than ‘leaner’ ones. We argue that strategies to counter ‘rippling’ may apply also to food supply systems, and furthermore observe, that many centralized food distribution networks in the westernized world resemble an hourglass-like network [30]. In fact, one may argue that it could for example apply to the European agri-food supply line, which consists of 11 Million farmers, of which 70% was smaller than 5 ha in 2010, who provide input for 0.3 Million of processing enterprises, who in turn sell to 2.8 Million distributing enterprises, who in turn sell to 500 Million consumers in the EU [43]. Resilience in agri-food supply chains is considered to be of increasing relevance [44]. It suggests we have to consider a trade-off between efficiency and resilience when dealing with questions on resilience, also on a larger, above-company scale. We argue below that this apparent trade-off may not be a straightforward reciprocal relation, but we emphasize here that our model results suggest one should take into account that increasing the resilience of a food chain in all likelihood may occur at the expense of efficiency, and *vice versa*.

A second remark is on the frequency, scale, and timing of shocks. In this paper we consider only one type of disruption per simulation. However, in reality there are different types of shock that can and do occur at different levels (near) simultaneously, including weather events affecting crop production, crises resulting from disease outbreaks (as demonstrated by the current COVID-19 pandemic), and prize volatility and other events affecting the traders and/or consumers. Also in the business economics literature it is remarked that risks should not be considered independently from other risks, and instead of putting the focus on individual disruption events, one should look at the gradual build-up of stress on a system [33]. This suggests that when considering the resilience of agri-food systems we should consider the joint effects of multiple shocks.

A third remark is on the choice of the methodology. The choice of modelling framework is relevant for the disaggregated description of flows between agents for calculating efficiency and resilience. Furthermore, by using an ABM, we explicitly represent the individual agents and their interactions, rather than some aggregation. The individual flows together serve as input for calculating the information-based criterion by Ulanowicz [42] we use in this paper as model output. The calculation of this output is dependent on the aggregation level of the flows. Without disaggregated flow input, the trade-off between efficiency and resilience does not appear—which does not mean it is not there. We argue one has to look at data at a disaggregated level, e.g. sub-regional and household levels, to explicitly expose such a trade-off. Currently, e.g., the FAOSTAT database [1] offers indicator statistics at these levels, but this information needs to be augmented with data on who trades with who. Another benefit of the information-based measure is that it can provide an estimation of resilience based on the network configuration without the need for shocks. It is in that sense a practical metric because it uses ‘snap-shot’ type of flow data, which is the typical type of available data on trading.

When using the information-based measure for quantifying resilience it needs to be considered that information can be expressed in different units, like carbon, nitrogen, water, or money. The results of the assessment may differ based on the units that are used. It is however important that resilience and efficiency are expressed in similar units to be able to make a comparison, i.e., one has to avoid comparing apples and oranges. Also, while the measure includes zero flows in the calculations, it does not distinguish between links that can potentially exist but do not at the moment (e.g., traders who could sell but do not by chance), and links that will probably never materialise (e.g., traders who never will sell because of other reasons). It explains the differences in the *A*-to-*B* (efficiency to resilience) ratios of the different interaction types, but also suggests that the utility of the measure is limited for social systems, where ‘soft’ boundaries resulting from human behaviour, decision making, and interactions can be expected. This is demonstrated by our simulations with the preference interaction mode, in which decisions by agents on whether or not to trade with another agent are not only based on optimizing economic utility, but also on trust. In those simulations the overall satisfaction was lower than in simulations with decisions based only on economic utility.

The information-based criterion was proposed by Ulanowicz in the context of food webs [42], where the nodes of the network are species rather than individuals. Food web models are typically represented by models of differential equations or system dynamics. For such systems, it has been shown that the existence of ‘weak’ links between many species (as compared to a few ‘strong’ links) stabilizes food webs [45]. Agri-food systems are not ecosystems of species, and an ABM is not a set of differential equations, yet our findings seem to roughly correspond to those for the species networks. If we define a ‘weak’ link as a trade link between two agents that on average exists only for a small part of the time window we look at, then networks with many of these ‘weak’ links (and hence many agents) seem to be the ‘stable’, or better, more resilient ones. Indeed, it has been argued that the current global food supply system is unstable and needs to be reorganized to increase its resilience against shocks, where an important role is envisaged for retailer parties [46]. Interestingly, there are currently European incentives to promote shorter food chains and alternative food networks [47], which may reduce the vulnerability of food systems. Our analysis suggests this could work, but at the cost of efficiency. The use of a measure like the information-based criterion could be helpful in distinguishing the resilience and efficiency of different potential food chain designs.

The current model focuses on primary mechanisms, which may limit utility for further research questions. First of all, there is no explicit modelling or monitoring of money. Though it does not seem to invalidate the type of dynamics we observe, there is no way of explicitly representing price volatility in the model. In addition, agents cannot go bankrupt and disappear from the system, and no new agents appear during simulations. One may argue that the values of aggregated variables like stocks, impact, *A*, and *B* may vary over the course of a simulation when agent replacement is allowed in the model. Second, there is no physical scale in the model. In reality agents for instance differ in the size of their farm and hence ability for generating crops. Also, no transport costs are included. Third, there is no turn-over in the model, and no technological progression of any sort. Fourth—and importantly—the diversity of agents of one type (producer, trader, consumer) is limited to the crops they hold or consume and possibly their trading history (in the case of preference interactions); there is yet no real diversity in the various properties of agents. Also, the agents in the current model are unrealistic, as they behave according to economic optimal principles (‘*Homo economicus*’) and display no further bounded rationality, social dynamics, capacity for learning, strategic thinking, or adaptation, while their objective functions are now considered only implicitly. While the agents in the current model can reorganize their trading network according to these economic principles and thus generate network resilience, it is not necessarily realistic agent behaviour. An additional simplification is that agents make only single links in this model *per simulation step*, while in reality they may have multiple links simultaneously. However, agents can make and break links across iterations, and we aggregate across these iterations, so we expect that the effect of this last simplification will be limited compared to some of the other above-mentioned model assumptions.

For future research, we foresee several expansions of the model. Importantly, relevant resilience-generating mechanisms are not yet covered. One mechanism that promotes resilience is heterogeneity in system components [48]; in particular diversity in agents would capture this. Another mechanism is buffer capacity. In the current version, crop item stocks are kept by traders, but other types of stocks can be included, such as monetary, time, and even technological capabilities. For instance, for producers it is key to maintain a livelihood [49], but arguably an agent has to earn considerably more to have space for making investments. In the current model economic utility is included implicitly. In future versions we aim to explicitly include price dynamics to simulate price shocks and restricted economic access to food. Also relevant is adaptation or learning by individuals, which in turn may result in adaptation on a system level. For example, in the current model, the preference interaction mode in which it is assumed that consumers lose their ‘trust’ very slowly may be oversimplified; in fact, this is slow adaptation. In reality, if a trader would continue to lose its stock, a consumer (and possibly also a producer) might quickly lose interest in trading with this agent, which constitutes a relevant type of learning. In the current model there is no replacement of agents during simulations. The addition of ‘deaths’ (bankruptcy and subsequent exit of agents) and ‘births’ (entry by new, competing agents, like producers or traders) may result in a system adapting to continuous shocks, for instance comparable to what was demonstrated in simulated populations where individual traits where inherited by offspring of high-fitness individuals (resilience through adaptation [50]). Properties like decision making (e.g., to decide on the production of cash crops), risk perception, bounded rationality, social dynamics, capacity for learning, strategic thinking, and individual adaptation can be captured by the explicit inclusion of objective functions involving ‘agent modules’ capable of conceptualizing and quantifying such processes, for instance as done by Consumat [51] or GRASP [52]. Future modifications of the presented model will focus on these aspects. Resilience is always a matter of “resilience of what to what, and for whom?”. By looking at explicit individual objectives of agents, one can also consider an explicit monitoring of the fulfilment of these individual objectives of each agent. This, in turn, may affect one’s assessment of resilience or efficiency. Another addition is the inclusion of the possibility that individual agents create multiple links in the same iteration, thus allowing for simultaneous trades with the same individual (where an individual could also be, e.g., a company or country).

Another topic for future research is the involvement of real-life data. The current model has not been calibrated or validated against real-life data. The FAOSTAT database [1] offers indicator statistics on national and lower (even household) levels. These data include information on region, area of residence, household size and composition, income groups, and groups by characteristics such as gender, age, education, economic activity, and occupation. Such data can be used for model ‘tuning’ based on states (i.e., the distributions of variables at fixed points in time). However, several important aspects are then still missing, such as data on who interacts or trades with who and why, or the decision-making processes. This implies that at least part of what makes resilience in a supply chain remains obscured or ‘invisible’, and the use of a model remains necessary to hypothesize about these elements.

## Conclusion

Summarizing, we use a dynamic model with simple principles of trade between agents and an information-based metric to assess the efficiency and resilience in stylized food supply chains. Certain network types together with trading according to economic rationality have a considerable level of throughput of food items (i.e., high efficiency), but at the same time also build up fewer stocks than alternative networks. Their lower number of potential relinkage options in case of disruption indicates a lower resilience against shocks. While these results seem to confirm a simple trade-off between resilience and efficiency, the trade-off is complicated by the trading interactions and the type of shock. The current model contains implicit objectives for individual agents, and in particular social aspects in, e.g., trading (like ‘trust’ between agents) may cause ‘soft’ boundaries or opportunities that may modify resilience and efficiency. Moreover, the trade-off may be modified by additional mechanisms for resilience that are now absent from the model. We argue the importance of considering there is a trade-off between resilience and efficiency when trying to reach the SDG2: zero hunger while also trying to improve the resilience of food supply systems to shocks resulting from, e.g., climate change, and advocate more research to further explore this trade-off and its ramifications for real-life food chains. Future developments should include expanding the presented framework in the economic (in particular explicit price dynamics), ecological (such as relevant biophysical food system aspects), and social domain (including social behaviour like bounded rationality), and matching the model framework to real-life applications involving data to further assess the reality of this trade-off.

## Supporting information

S1 Fig One example of a simulation run time series with a sliding window. Calculated are Eq (2), the stocks, and *A* and *B* from Eq (4), using a sliding window of five, i.e., at each production cycle (or ‘simulation year’) the average is taken over the values of the five consecutive production cycles. The right period is the pre-shock phase (in red); the middle period is the shock phase (in blue); the left period is the post-shock phase (in green), added as validation that the values are restored to pre-shock levels. The pre- and post-shock periods display roughly the same averaged values for all variables. This example concerns a small network (five agents of each type). As a result, the ‘noise’ is considerable. Nevertheless, these results suggest it may be reasonable to take the average across the whole period as approximation. Note, that under different model assumptions, the dynamics may be changing over time, in which case taking a sliding window is a better option that averaging.
(TIF)

S2 Fig A second example of a simulation run time series with a sliding window. Similar to S1 Fig. This example concerns a larger network (twenty agents of each type). As a result, the ‘noise’ is much less than in the other example. Also, *A* and *B* are higher because more agents are included in the network.
(TIF)
