# Supplementary figures and images for: An agent based model representation to assess resilience and efficiency of food supply chains
Source: PLoS One. 2020 Nov 19;15(11):e0242323. doi: 10.1371/journal.pone.0242323 (PMC7676680; doi:10.1371/journal.pone.0242323)

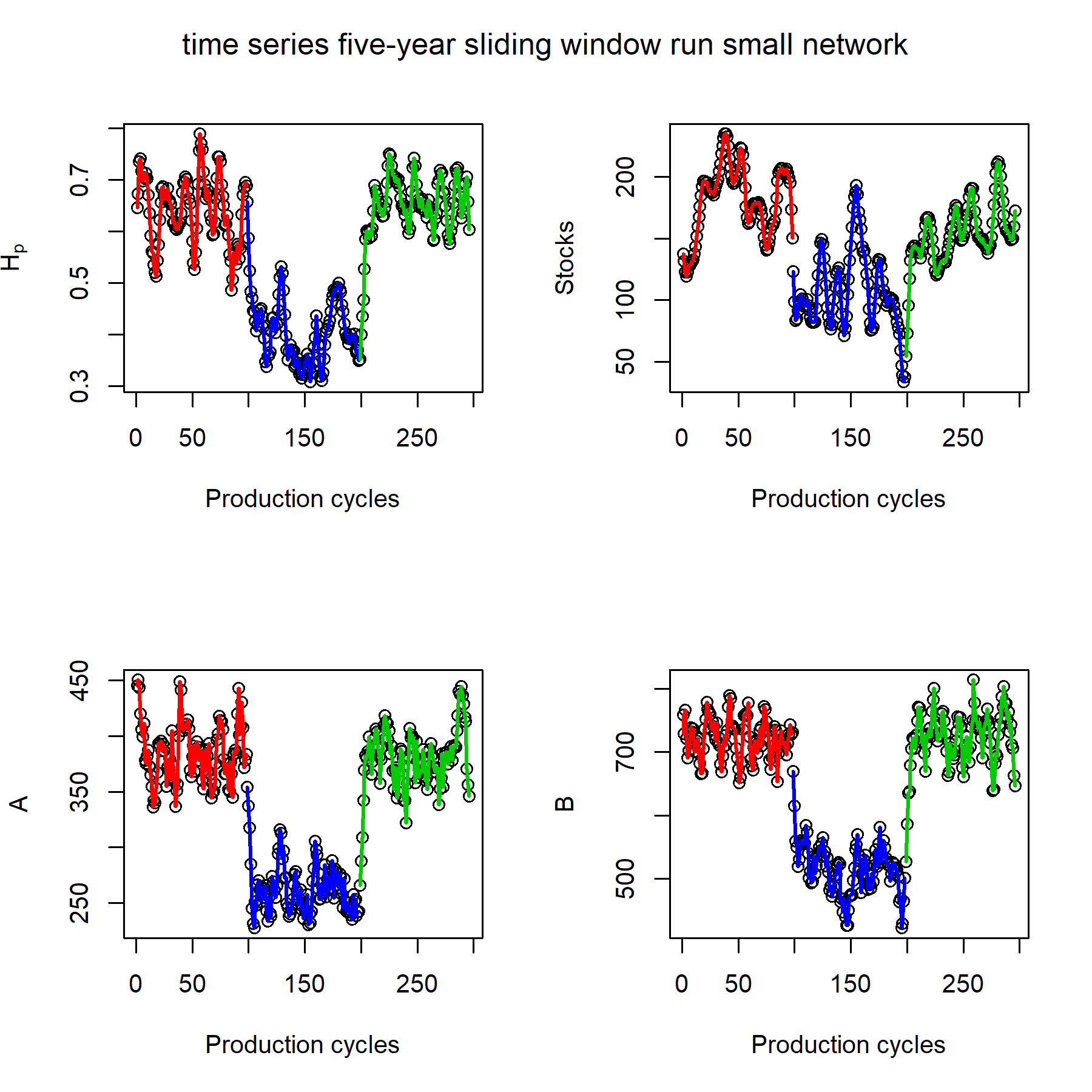

Supplement: S1 Fig — Calculated are Eq (2), the stocks, and A and B from Eq (4), using a sliding window of five, i.e., at each production cycle (or ‘simulation year’) the average is taken over the values of the five consecutive production cycles. The right period is the pre-shock phase (in red); the middle period is the shock phase (in blue); the left period is the post-shock phase (in green), added as validation that the values are restored to pre-shock levels. The pre- and post-shock periods display roughly the same averaged values for all variables. This example concerns a small network (five agents of each type). As a result, the ‘noise’ is considerable. Nevertheless, these results suggest it may be reasonable to take the average across the whole period as approximation. Note, that under different model assumptions, the dynamics may be changing over time, in which case taking a sliding window is a better option that averaging. (TIF) [file pone.0242323.s001.tif]

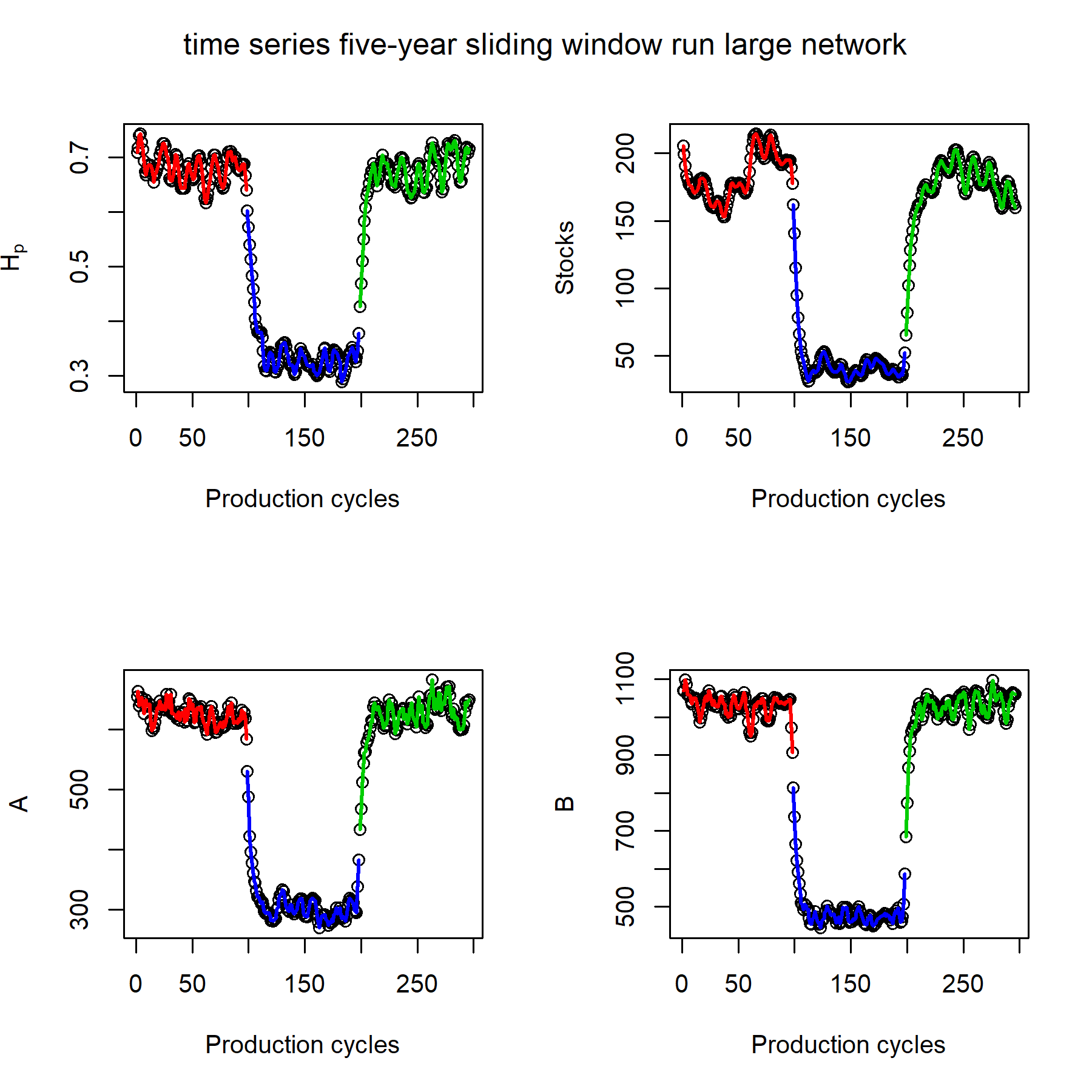

Supplement: S2 Fig — Similar to S1 Fig. This example concerns a larger network (twenty agents of each type). As a result, the ‘noise’ is much less than in the other example. Also, A and B are higher because more agents are included in the network. (TIF) [file pone.0242323.s002.tif]
